# Knowledge and practice of hemodialysis catheter care and associated factors among patients on maintenance hemodialysis: An analytical cross-sectional study

**DOI:** 10.1371/journal.pone.0353737

**Published:** 2026-07-14

**Authors:** Maheda Jilisha Lucas, Emmanuel Sumari, Joel Seme Ambikile

**Affiliations:** 1 Bugando Medical Centre, Mwanza, Tanzania; 2 Department of Clinical Nursing, Muhimbili University of Health and Allied Sciences, Dar es Salaam, Tanzania; Japanese Red Cross Medical Center, JAPAN

## Abstract

**Background:**

Effective care of hemodialysis catheters (HDCs) is essential for preventing complications, particularly catheter-related infections, among patients receiving maintenance hemodialysis. However, gaps in patient knowledge and practice may compromise optimal catheter care. This study assessed the level of knowledge and practice of HDC care and identified factors associated with these outcomes among patients on maintenance hemodialysis.

**Methods:**

A cross-sectional study was conducted among 97 patients undergoing maintenance hemodialysis. Data were collected using a structured questionnaire assessing socio-demographic and clinical characteristics, as well as knowledge and practice related to HDC care. Knowledge and practice scores were categorized using an 80% cutoff. Bivariate and multivariable logistic regression analyses were performed to identify factors associated with knowledge and practice. Model fitness and multicollinearity were assessed.

**Results:**

Overall, 51.5% of respondents demonstrated satisfactory knowledge, while 45.4% reported satisfactory HDC care practices. Knowledge was highest regarding the purpose and insertion site of the catheter but lower for aspects related to infection recognition and dressing care. In multivariable analysis, female sex (AOR: 3.09; 95%CI: 1.12, 8.54; *p* = 0.030) and shorter duration on hemodialysis (<2 years) (AOR: 3.66; 95%CI: 1.28, 10.50; *p* = 0.016) were independently associated with satisfactory knowledge. Regarding practice, knowledge emerged as the only independent predictor, with respondents having satisfactory knowledge demonstrating significantly higher odds of satisfactory practice (AOR: 47.39; 95%CI: 11.14, 201.64; *p* < 0.001). Other variables were not significantly associated with practice after adjustment.

**Conclusion:**

Less than half of patients demonstrated satisfactory HDC care practices despite moderate levels of knowledge. Knowledge was strongly associated with practice, highlighting its potential importance in supporting optimal catheter care behaviors. These findings indicate that targeted and sustained patient education may be important in improving knowledge and supporting better catheter care practices. Further multicenter and longitudinal studies using more robust measurement approaches are recommended to better clarify factors influencing HDC care practices and related outcomes.

## Introduction

Chronic kidney disease (CKD) is a major global public health problem, affecting approximately 850 million people worldwide, with a prevalence estimated at 11–13%, predominantly in low- and middle-income countries (LMICs) [1–3]. As CKD progresses to end-stage renal disease (ESRD), renal replacement therapy (RRT), including hemodialysis, becomes life-sustaining. Hemodialysis is the most commonly used modality of RRT, involving extracorporeal removal of waste products and excess fluid from the blood [4,5].

Globally, between 4.9 and 9.7 million individuals require RRT, with the majority residing in LMICs where access to optimal care remains limited [6]. Due to late presentation, financial constraints, and limited availability of permanent vascular access, approximately 60% of patients initiate hemodialysis using central venous catheters [7]. Although these catheters provide rapid vascular access, they are associated with serious complications including catheter-related infections, thrombosis, and mechanical dysfunction, contributing significantly to morbidity and mortality [8,9].

Evidence shows that inadequate patient knowledge and poor catheter care practices contribute significantly to complications in dialysis care. Many patients receive insufficient education and have limited understanding of dialysis and catheter management, which negatively affects self-care and adherence to preventive measures [10]. Research evaluating patient education in dialysis care has highlighted gaps in knowledge and comprehension, while studies on knowledge, attitudes, and practices among hemodialysis patients report that such deficits are associated with suboptimal practices and poorer outcomes [11,12]. These deficiencies increase the risk of infection, prolong hospital stays, and worsen dialysis outcomes, underscoring the need for strengthened patient education and support.

In Tanzania, community-based studies estimate the prevalence of chronic kidney disease (CKD) to range from 7.0% to 12.4%. A study in Northern Tanzania reported an overall prevalence of 7.0%, with a higher burden among urban residents (15.2%) compared to rural populations (2.0%) [13], while a rural study in Kisarawe district reported a prevalence of 12.4% for CKD stages III–V [14], indicating a substantial burden across both settings. The number of patients requiring hemodialysis is increasing, with approximately 3,231 individuals currently receiving therapy nationwide [15,16]. Over 60% of these patients rely on hemodialysis catheters due to late presentation and limited vascular access options [17]. Consequently, catheter-related infections are common, with some Tanzanian studies reporting rates exceeding 50%, alongside complications such as dysfunction, thrombosis, central venous stenosis, and dislodgement [18].

Despite national guidelines emphasizing infection prevention and aseptic catheter care practices [19], alongside the expansion of dialysis services and improved access to care in Tanzania [20], limited evidence exists on patients’ knowledge and self-care practices related to hemodialysis catheter (HDC) care. This gap is critical, as inadequate knowledge and suboptimal practices may contribute to preventable complications, including catheter-related infections. Therefore, this study aimed to assess the knowledge and practices of hemodialysis catheter care and determine associated factors among patients at Bugando Medical Centre (BMC). The findings are expected to inform targeted interventions to improve patient outcomes and reduce catheter-related complications in Tanzania.

## Methods

### Study design

An analytical cross-sectional study design was applied to determine the prevalence of knowledge and practice regarding hemodialysis catheter care and to identify associated factors at a single point in time [21,22]. While appropriate for estimating associations, this design does not allow for causal inference or determination of temporal relationships between knowledge and practice.

### Study setting

This study was conducted at Bugando Medical Centre (BMC) in Mwanza Region, northwestern Tanzania. BMC is a tertiary referral and teaching hospital, established in 1971 and jointly owned by the Government of Tanzania and the Catholic Church through the Tanzania Episcopal Conference. It serves as the main referral center for the Lake Zone and surrounding regions, providing healthcare services to over 14 million people across eight regions. The hospital has an approximate bed capacity of 900 and offers a wide range of general and specialized services, including internal medicine, surgery, pediatrics, obstetrics and gynecology, oncology, and intensive care.

The renal unit at BMC is equipped with 16 Fresenius 5008S hemodialysis machines and staffed by two nephrologists, two registrars, one nephrology nurse, and nine additional nurses. Each dialysis session lasts approximately four hours, with two main sessions conducted daily (morning and afternoon), and occasional evening sessions. This setup allows the unit to serve approximately 30–40 patients per day. Patients typically undergo two to three dialysis sessions per week depending on their clinical condition. Over a two-week period, the unit serves approximately 112 unique patients without repetition.

BMC was selected as the study site due to its status as the largest tertiary referral hospital in the Lake Zone, serving a diverse patient population. Its well-established renal unit and consistent provision of hemodialysis services made it an appropriate setting for reliable data collection, and the findings are likely to be generalizable to similar settings within the region.

### Study population and eligibility criteria

The study population comprised adult patients (aged ≥18 years) with hemodialysis catheters (both tunneled and non-tunneled central venous catheters) who were undergoing hemodialysis treatment at BMC in Mwanza Region. Eligible participants were those who had been on hemodialysis for at least one month, were able to communicate effectively, and were willing to provide informed consent. Patients who were critically ill, had cognitive impairments that limited their ability to respond to the questionnaire, or declined to participate were excluded from the study.

### Sample size and sampling procedure

A total population sampling approach was employed in this study due to the relatively small and known number of patients undergoing hemodialysis at the BMC renal unit. The unit had approximately 112 patients receiving hemodialysis during the study period; therefore, all eligible patients were considered for inclusion in the study. Participants were recruited using a consecutive sampling technique, whereby all eligible patients who attended the renal unit during the data collection period and met the inclusion criteria were invited to participate until the entire accessible population was covered.

### Data collection tool

Data were collected between 26 May and 28 June 2025 using a self-administered questionnaire with closed-ended questions. The tool was adapted from previous studies to assess factors associated with knowledge and practice regarding HDC care among patients [12,23]. To ensure maximum participation, the questionnaire was translated from English into Swahili, the primary language of most respondents. The instrument was organized into three sections in line with the study objectives. The first section collected sociodemographic information, including age, sex, marital status, education level, employment status, place of residence, vascular access type, number of weekly HD sessions, and duration of hemodialysis. The second section assessed respondents’ knowledge of hemodialysis catheters, including their understanding of catheter care and related complications. The third section evaluated patients’ practices regarding HDC care.

To ensure content validity, the data collection tool was reviewed by a nephrologist and a nephrology nurse working in the renal unit at BMC to assess the clarity, relevance, and adequacy of the items in measuring the intended constructs. The questionnaire was then pre-tested among five patients with similar characteristics to the study population to evaluate its clarity and feasibility, and data obtained from this pre-test were not included in the final analysis. The reliability analysis yielded a Cronbach’s alpha of 0.896 for all items scale, indicating good internal consistency. Although the questionnaire demonstrated good internal consistency and underwent expert review for content relevance, further psychometric assessment of construct validity was not performed.

Due to the high risk to patient health and the critical importance of hemodialysis catheter care, a cutoff of ≥80% was used to define satisfactory knowledge and practice. This threshold aligns with operational definitions commonly applied in Knowledge, Attitude, and Practice (KAP) studies conducted in Africa, particularly in Egypt, where a ≥ 80% benchmark has been used in infection prevention and clinical patient care research. [24,25].

Knowledge and practice regarding hemodialysis catheter (HDC) care were conceptualized as related but distinct constructs. Knowledge referred to the patient’s understanding of recommended hemodialysis catheter care procedures and prevention of complications, while practice referred to the self-reported application of these recommendations in daily catheter care behaviors. Although distinct, these constructs may overlap in measurement, particularly in self-reported assessments. Knowledge was assessed using 12 closed-ended questions. Each correct response was assigned one point, while incorrect responses received zero, yielding a maximum possible score of 12. The total score was converted into a percentage, and respondents scoring ≥80% were classified as having satisfactory knowledge, whereas those scoring <80% were considered to have unsatisfactory knowledge. Practice related to HDC care was assessed using 15 Likert-scale items ranging from “Never” (1) to “Always” (5), with a maximum possible score of 60. The total score was converted into a percentage, and respondents scoring ≥80% were categorized as having satisfactory practice, while those scoring <80% were classified as having unsatisfactory practice.

### Data collection procedure

Participants for the study were approached by the researcher and research assistant (holder of bachelor of science in nursing) at the renal unit and during clinic visits at BMC. The researchers explained the study’s purpose, benefits, potential risks, and eligibility criteria to eligible patients and obtained their informed consent for participation. Those who agreed were provided with a written consent form to sign voluntarily prior to enrolment. The research assistant was first trained and familiarized with the study procedures before commencement of data collection.

The questionnaire, which took approximately 30 minutes to complete, was self-administered by participants during hemodialysis sessions and clinic visits within the renal unit. Data collection during hemodialysis was conducted after the first 30–60 minutes of the session, when patients were clinically stable and comfortable, to avoid interference with treatment and ensure patient safety. For a few participants who required assistance due to difficulty in reading or filling in the questionnaire, support was provided by the researcher and trained research assistants. The researcher and research assistants ensured anonymity, privacy, and respectful communication throughout the data collection process in a private environment to create a comfortable setting and enhance response rates.

### Data analysis

Data were checked for completeness and consistency, then coded and entered into Statistical Package for the Social Sciences (SPSS) version 25 for analysis. Descriptive statistics were used to summarize respondents’ characteristics, knowledge, and practice regarding HDC care. Categorical variables were presented as frequencies and percentages. Bivariate logistic regression analysis was performed to assess the association between independent variables (sociodemographic and clinical characteristics) and the outcomes (knowledge and practice of HDC care). Variables with p-values less than 0.2 in the bivariate analysis were included in the multivariable logistic regression models to identify independent predictors of knowledge and practice. Adjusted odds ratios (AOR) with 95% confidence intervals (CI) were reported. Model fitness was assessed using the Hosmer-Lemeshow goodness-of-fit test, and multicollinearity among independent variables was evaluated using variance inflation factor (VIF) and tolerance values. Statistical significance was set at a *p*-value of less than 0.05 for all analyses.

### Ethical considerations

Ethical approval for this study was obtained from the Institutional Review Board of Muhimbili University of Health and Allied Sciences (MUHAS), with approval number MUHAS-REC-04-2025-2896. Permission to conduct the study was also obtained from the management of BMC prior to the commencement of data collection. Participation in the study was voluntary. All participants were provided with detailed information regarding the study objectives, procedures, potential risks, and benefits, and were given the opportunity to make an informed decision about their participation. Written informed consent was obtained from each participant prior to data collection. Participants were also informed of their right to withdraw from the study at any time without any consequences. Confidentiality and anonymity were strictly maintained throughout the study. No personal identifiers were recorded on the questionnaires, and all information collected was handled with strict confidentiality.

## Results

### Respondents’ socio-demographic and clinical characteristics

A total of 112 eligible patients were approached to participate in the study. Of these, 97 consented and completed the questionnaire, yielding a response rate of 86.6%. These participants were included in the final analysis. More than half were aged above 40 years (n = 54, 55.7%), while 43 (44.3%) were aged 40 years and below. The majority were male (n = 61, 62.9%), and 36 (37.1%) were female. Regarding marital status, most respondents were in a partnership (n = 62, 63.9%), while 35 (36.1%) were non-partnered. Slightly more than half had attained secondary education or higher (n = 54, 55.7%), whereas 43 (44.3%) had primary education or lower. In terms of employment, 55 (56.7%) were employed, while 42 (43.3%) were unemployed. The majority resided in rural areas (n = 84, 86.6%), with 13 (13.4%) residing in urban settings.

Clinically, most respondents were using permanent catheters (Permcath) (n = 56, 57.7%), while 41 (42.3%) had temporary catheters (tempcath). Most patients received hemodialysis three times per week (n = 67, 69.1%), while 30 (30.9%) had two sessions weekly. More than half had been on hemodialysis for less than two years (n = 61, 62.9%), while 36 (37.1%) had a duration of two years or more. Table 1 summarizes the socio-demographic and clinical characteristics of respondents.

**Table 1:** Socio-demographic and clinical characteristics of respondents (n = 97).

Variable	Category	Frequency (n)	Percent (%)
Age	Up to 40 years	43	44.3
	> 40 years	54	55.7
Sex	Male	61	62.9
	Female	36	37.1
Marital status	Non-partnered	35	36.1
	Partnered	62	63.9
Education level	Primary or lower	43	44.3
	Secondary or higher	54	55.7
Employment status	Unemployed	42	43.3
	Employed	55	56.7
Residence	Urban	13	13.4
	Rural	84	86.6
Vascular access type	Tempcath	41	42.3
	Permcath	56	57.7
Number of weekly sessions	2 sessions	30	30.9
	3 sessions	67	69.1
Duration of hemodialysis	< 2 years	61	62.9
	≥ 2 years	36	37.1

### Respondents’ knowledge regarding HDC care

Among the respondents, 50 (51.5%) demonstrated satisfactory knowledge of hemodialysis catheter (HDC) care. Knowledge levels varied across individual items. The highest proportions of correct responses were observed for the primary purpose of a hemodialysis catheter, correctly identified by 90 (92.8%) respondents, and the common site of catheter insertion, correctly identified by 85 (87.6%) respondents. In contrast, the lowest proportions of correct responses were reported for the recommended frequency of catheter dressing change, correctly identified by 65 (67.0%) respondents, and appropriate actions to take when redness, swelling, or pus are observed at the catheter site, correctly identified by 70 (72.2%) respondents. Table 2 summarizes the knowledge scores for HDC care.

**Table 2:** Respondents responses regarding knowledge on hemodialysis catheter care.

	Question	Correct(n)	%	Incorrect(n)	%
1.	What is the primary purpose of a hemodialysis catheter?	90	92.8	7	7.2
2.	Where a hemodialysis catheter is commonly inserted?	85	87.6	12	12.4
3.	Which of the following is the correct way to keep your catheter site clean?	76	78.4	21	21.6
4.	Why is it important not to get the catheter site wet?	72	74.2	25	25.8
5.	How often should the catheter dressing be changed?	65	67.0	32	33.0
6.	What should you do if you notice redness, swelling, or pus at the catheter site?	70	72.2	27	27.8
7.	Can you shower with a hemodialysis catheter?	76	78.4	21	21.6
8.	Why should you avoid using the catheter arm for blood pressure checks or blood draws?	71	73.2	26	26.8
9.	What is a sign of catheter malfunction during dialysis?	77	79.4	20	20.6
10.	Who is responsible for caring for your catheter site during dialysis?	74	76.3	23	23.7
11.	What can happen if the catheter is not cared for properly?	81	83.5	16	16.5
12.	Why should you avoid lifting heavy objects with the catheter arm?	71	73.2	26	26.8

### Factors associated with respondents’ knowledge regarding HDC care

Table 3 shows the bivariate and multivariable logistic regression analyses of factors associated with knowledge of HDC care. In the bivariate logistic regression analysis, several factors were significantly associated with knowledge of HDC care. Respondents aged >40 years had lower odds of satisfactory knowledge compared to those aged ≤40 years (COR: 0.35, 95%CI: 0.15, 0.80; *p* = 0.013). Female respondents had higher odds of satisfactory knowledge compared to males (COR: 2.73; 95%CI: 1.16, 6.40; *p* = 0.021). Higher education level was marginally associated with satisfactory knowledge (COR: 2.27; 95%CI: 1.00, 5.17; *p* = 0.050). Employed respondents were more likely to have satisfactory knowledge compared to unemployed respondents (COR: 3.61; 95%CI: 1.54, 8.46; *p* = 0.003). Respondents with a permanent catheter had higher odds of satisfactory knowledge compared to those with a temporary catheter (COR: 4.03; 95%CI: 1.70, 9.55; *p* = 0.002). Additionally, respondents with a shorter duration on hemodialysis (<2 years) were more likely to have satisfactory knowledge compared to those with ≥2 years (COR: 3.30; 95%CI: 1.39, 7.85; *p* = 0.007). Marital status, residence, and dialysis frequency were not significantly associated with knowledge.

**Table 3:** Bivariate and multivariable logistic regression analysis of factors associated with knowledge of HDC care.

		Unsatisfactory		Satisfactory					
Variable	Category	n	%	n	%	COR (95% CI)	*p*-value	AOR (95% CI)	*p*-value
Age	≤40	16	37.2	27	62.8	1.00 (Ref)		1.00 (Ref)	
	>40	34	63.0	20	37.0	0.35 (0.15, 0.80)	0.013	0.49 (0.14, 1.72)	0.265
Sex	Male	37	60.7	24	39.3	1.00 (Ref)		1.00 (Ref)	
	Female	13	36.1	23	63.9	2.73 (1.16, 6.40)	0.021	3.09 (1.12, 8.54)	0.030
Marital status	Non-partnered	20	57.1	15	42.9	1.00 (Ref)		–	
	Partnered	30	48.4	32	51.6	1.42 (0.62, 3.28)	0.408	–	–
Education	Primary or lower	27	62.8	16	37.2	1.00 (Ref)		1.00 (Ref)	
	Secondary /higher	23	42.6	31	57.4	2.27 (1.00, 5.17)	0.050	0.68 (0.21, 2.15)	0.507
Employment	Unemployed	29	69.0	13	31.0	1.00 (Ref)		1.00 (Ref)	
	Employed	21	38.2	34	61.8	3.61 (1.54, 8.46)	0.003	3.17 (0.86, 11.67)	0.084
Residence	Rural	8	61.5	5	38.5	1.00 (Ref)		–	
	Urban	42	50.0	42	50.0	1.60 (0.48, 5.29)	0.441	–	–
Vascular access	Tempcath	29	70.7	12	29.3	1.00 (Ref)		1.00 (Ref)	
	Permcath	21	37.5	35	62.5	4.03 (1.70, 9.55)	0.002	2.50 (0.90, 6.94)	0.079
Dialysis frequency	2 sessions	34	50.7	33	49.3	1.00 (Ref)		–	
	3 sessions	16	53.3	14	46.7	0.90 (0.38, 2.14)	0.814	–	–
Duration on HD	≥2 years	38	62.3	23	37.7	1.00 (Ref)		1.00 (Ref)	
	<2 years	12	33.3	24	66.7	3.30 (1.39, 7.85)	0.007	3.66 (1.28, 10.50)	0.016

In the multivariable logistic regression analysis, female sex and shorter duration on hemodialysis remained independently associated with knowledge of HDC care. Female respondents had higher odds of satisfactory knowledge compared to males (AOR: 3.09; 95%CI: 1.12, 8.54, *p* = 0.030). Similarly, respondents with <2 years on hemodialysis were more likely to have satisfactory knowledge compared to those with ≥2 years (AOR: 3.66; 95%CI: 1.28, 10.50; *p* = 0.016). Although not statistically significant, higher odds of satisfactory knowledge were observed among employed respondents (AOR: 3.17; 95%CI: 0.86, 11.67; *p* = 0.084) and those with a permanent catheter (AOR: 2.50; 95%CI: 0.90, 6.94; *p* = 0.079). Age and education level were not significantly associated with knowledge after adjustment.

The Hosmer and Lemeshow test indicated an acceptable model fit (χ² = 12.784, df = 7, *p* = 0.078), showing no significant difference between observed and predicted values, and suggesting that the model fits the data adequately. Similarly, the multicollinearity test showed no evidence of significant multicollinearity among the independent variables. All tolerance values were >0.10 (0.472–0.904), and all VIF values ranged from 1.106 to 2.119, which are below the conservative cutoff of 5. This indicates that the predictors were not highly correlated, and the regression estimates are stable.

## Respondents’ practice regarding HDC care

Overall, 44 (45.4%) respondents demonstrated satisfactory practice regarding HDC care, although practices varied across individual items. The highest satisfactory practice levels were observed in avoiding swimming or soaking the body in water, reported by 80 (82.5%) respondents, and avoiding wearing tight clothing over the catheter site, reported by 64 (66.0%) respondents. In contrast, the lowest satisfactory practice levels were noted in reporting immediately if the catheter site is red, swollen, or has discharge, reported by 30(30.9) respondents, and avoiding touching the catheter site unless it is necessary, reported by 36(37.1) respondents. Table 4 summarizes the practice scores for HDC care.

**Table 4:** Respondents’ practice scores regarding HDC care.

	Questions	Never n (%)	Rarely n (%)	Sometimes n (%)	Often n (%)	Always n (%)
1.	Do you wash your hands with soap and water before touching your catheter area?	5(5.2)	20(20.6)	14(14.4)	18(18.6)	40(41.2)
2.	Do you use hand sanitizer when soap and water are not available before touching your catheter?	16(16.5)	12(12.4)	16(16.5)	12(12.4)	41(42.3)
3.	Do you avoid touching your catheter site unless it is necessary	3 (3.1)	10(10.3)	22(22.7)	26(26.8)	36(37.1)
4.	Do you keep your catheter site clean and dry at all times	3(3.1)	7(7.2)	15(15.5)	29(29.9)	43(44.3)
5.	Do you apply creams or ointments to your catheter site without a doctor’s instruction?	28(28.9)	3(3.1)	14(14.4)	11(11.3)	41(42.3)
6.	Do you change your catheter dressing regularly as advised by healthcare providers	31(32.0)	6(6.2)	4(4.1)	13(13.4)	43(44.3)
7.	Do you report immediately if your catheter site is red, swollen, or has discharge?	6(6.2)	4(4.1)	18(18.6)	39(40.2)	30(30.9)
8.	Do you cover your catheter site to keep it dry when bathing or showering	9(9.3)	13(13.4)	11(11.3)	19(19.6)	45(46.4)
9.	Do you avoid swimming or soaking your body in water (e.g., tubs or ponds)?	2(2.1)	2(2.1)	2(2.1)	11(11.3)	80(82.5)
10.	Do you avoid wearing tight clothing over the catheter site?	2(2.1)	2(2.1)	13(13.4)	15(15.5)	64(66.0)
11.	Do you come to dialysis sessions with a clean and dry catheter dressing?	2(2.1)	2(2.1)	15(15.5)	29(29.9)	49(50.5)
12.	Do you follow all instructions given by the dialysis staff during your treatment?	1(1.0)	2(2.1)	13(13.4)	32(33.0)	49(50.5)
13.	Do you tell the nurse or doctor if you feel pain or discomfort around your catheter during dialysis?	2(2.1)	3(3.1)	21(21.6)	31(32.0)	40(41.2)
14.	Do you know the signs of infection or problems around your catheter (e.g., fever, redness, and swelling)?	20(20.6)	11(11.3)	11(11.3)	16(16.5)	39(40.2)
15.	Do you report fever, chills, or other symptoms that might suggest a catheter infection?	0(0.0)	6(6.2)	11(11.3)	37(38.1)	43(44.3)

### Factors associated with respondents’ practice regarding HDC care

In the bivariate logistic regression analysis, employment status and vascular access type were significantly associated with the practice of hemodialysis catheter (HDC) care. Employed respondents had higher odds of demonstrating satisfactory practice compared to unemployed respondents (COR: 2.40; 95%CI: 1.04, 5.52; *p* = 0.039). Similarly, respondents with a permanent catheter were more likely to report satisfactory practices than those with a temporary catheter (COR: 4.79; 95%CI: 1.96, 11.69; *p* = 0.001). Notably, respondents with satisfactory knowledge had markedly higher odds of demonstrating satisfactory HDC care practices compared to those with unsatisfactory knowledge (COR: 43.88; 95%CI: 13.26, 145.21, *p* < 0.001). Female sex (COR: 2.31; 95%CI: 1.00–5.36; *p* = 0.051) and shorter duration on hemodialysis (<2 years) (COR: 2.31; 95%CI: 1.00, 5.36; *p* = 0.051) demonstrated borderline associations with satisfactory practice. In contrast, age, marital status, education level, residence, and dialysis frequency were not significantly associated with HDC care practices.

In the multivariable logistic regression analysis, only knowledge remained significantly associated with HDC care practice. Respondents with satisfactory knowledge had higher odds of satisfactory practice compared to those with unsatisfactory knowledge (AOR: 47.39; 95% CI: 11.14, 201.64; *p* < 0.001). Other variables, including age, sex, employment status, vascular access type, and duration of hemodialysis, were not significantly associated with practice after adjustment. The Hosmer and Lemeshow test indicated a good model fit (χ² = 4.649, df = 7, *p* = 0.703), suggesting no significant difference between observed and predicted values. Multicollinearity diagnostics showed no evidence of collinearity among predictors, with tolerance values ranging from 0.494 to 0.878 (>0.10) and VIF values from 1.139 to 2.023 (<5), indicating stable regression estimates Table 5.

**Table 5:** Bivariate and multivariable logistic regression analysis of factors associated with practice of HDC care.

		Unsatisfactory		Satisfactory					
Variable	Category	n	%	n	%	COR (95% CI)	*p*-value	AOR (95% CI)	*p*-value
Age	≤40	20	46.5	23	53.5	1.00 (Ref)		1.00 (Ref)	
	>40	33	61.1	21	38.9	0.55 (0.25, 1.25)	0.153	1.59 (0.30, 8.33)	0.582
Sex	Male	38	62.3	23	37.7	1.00 (Ref)		1.00 (Ref)	
	Female	15	41.7	21	58.3	2.31 (1.00, 5.36)	0.051	1.27 (0.35, 4.65)	0.718
Marital status	Non-partnered	21	60.0	14	40.0	1.00 (Ref)		–	
	Partnered	32	51.6	30	48.4	1.41 (0.61, 3.26)	0.426	–	–
Education	Primary or lower	26	60.5	17	39.5	1.00 (Ref)		–	
	Secondary /higher	27	50.0	27	50.0	1.53 (0.68, 3.44)	0.305	–	–
Employment	Unemployed	28	66.7	14	33.3	1.00 (Ref)		1.00 (Ref)	
	Employed	25	45.5	30	54.5	2.40 (1.04, 5.52)	0.039	0.89 (0.17, 4.69)	0.893
Residence	Rural	9	69.2	4	30.8	1.00 (Ref)		–	
	Urban	44	52.4	40	47.6	2.05 (0.58, 7.16)	0.263	–	–
Vascular access	Tempcath	31	75.6	10	24.4	1.00 (Ref)		1.00 (Ref)	
	Permcath	22	39.3	34	60.7	4.79 (1.96, 11.69)	0.001	3.40 (0.93, 12.47)	0.065
Dialysis frequency	2 sessions	36	53.7	31	46.3	1.00 (Ref)		–	
	3 sessions	17	56.7	13	43.3	0.89 (0.37, 2.11)	0.788	–	–
Duration on HD	≥2 years	38	62.3	23	37.7	1.00 (Ref)		1.00 (Ref)	
	<2 years	15	41.7	21	58.3	2.31 (1.00, 5.36)	0.051	0.65 (0.17, 2.50)	0.528
Knowledge on HDC care	Unsatisfactory	45	90.0	5	10.0	1.00 (Ref)			
	Satisfactory	8	17.0	39	83.0	43.88 (13.26, 145.21	<0.001	47.39 (11.14, 201.64)	<0.001

## Discussion

This study assessed knowledge and practice of HDC care and identified factors associated with these outcomes among patients receiving maintenance hemodialysis. Overall, approximately half of the respondents demonstrated satisfactory knowledge, while slightly fewer reported satisfactory practices, indicating suboptimal adherence to recommended catheter care behaviors. Importantly, knowledge emerged as the strongest factor associated with practice, with individuals possessing satisfactory knowledge being substantially more likely to demonstrate appropriate HDC care practices. These findings suggest that while basic understanding of catheter care exists among patients, gaps remain in translating knowledge into consistent practice, highlighting the need for strengthened patient education and reinforcement strategies within dialysis care programs.

The level of knowledge observed is moderate and consistent with findings from similar settings, where patients tend to demonstrate adequate understanding of general concepts but gaps in practical aspects of care. In this study, knowledge was highest for fundamental items such as the purpose and insertion site of the catheter, but lower for key preventive practices, including dressing change frequency and appropriate responses to signs of infection. This pattern has been widely reported, suggesting that while initial education may be effective in conveying basic information, it is often insufficient for ensuring comprehensive understanding of day-to-day catheter care [26,27]. These gaps are clinically significant, as inadequate knowledge has been linked to increased risk of catheter-related complications, including infections [28].

Importantly, this study identified female sex and shorter duration on hemodialysis (<2 years) as independent predictors of satisfactory knowledge. Female respondents were more likely to have adequate knowledge compared to males, which is consistent with evidence suggesting that women are generally more likely to engage in health-seeking behaviors and utilize healthcare services, potentially leading to greater exposure to health-related information [29]. This may reflect gender differences in health literacy, patient-provider communication, and adherence to treatment and educational recommendations, which are known to influence patients’ understanding and engagement in care [30].

Similarly, respondents with a shorter duration on hemodialysis demonstrated higher levels of knowledge compared to those on long-term treatment. This finding is particularly noteworthy, as it suggests a potential decline in knowledge retention over time. Patients newly initiated on dialysis are more likely to receive structured and intensive education at the start of treatment; however, evidence suggests that ongoing reinforcement and follow-up education are often required to sustain knowledge and adherence over time, and may diminish if not systematically maintained [27]. This underscores the need for continuous, rather than one-time, patient education strategies to sustain knowledge over the course of chronic therapy [31,32].

Although employment status and vascular access type were significantly associated with knowledge in the bivariate analysis, these associations did not persist after adjustment, although borderline trends remained. Employment status may influence knowledge of hemodialysis catheter care, as socioeconomic factors are strongly associated with health literacy, access to health information, and engagement with healthcare services [33]. Additionally, patients with permanent catheters may demonstrate better HDC care knowledge, potentially due to more frequent contact with healthcare providers and repeated exposure to counseling during ongoing care [27,31] This suggests that their effects may be partially mediated by other factors, including access to information and interaction with healthcare providers. However, since these associations were not statistically significant in the adjusted analysis, they should be interpreted with caution.

Practice of HDC care was suboptimal, with less than half of respondents demonstrating satisfactory behaviors. While adherence was high for certain practices such as avoiding water exposure, critical deficiencies were observed in infection-related behaviors, including prompt reporting of symptoms and minimizing unnecessary catheter manipulation. These findings are concerning, as such practices are essential for preventing catheter-related bloodstream infections. The discrepancy between knowledge and practice scores observed in this study is consistent with existing literature, which highlights that knowledge alone is insufficient to ensure behavior change, and that additional behavioral and system-level factors must be addressed [34–36].

A key finding of this study is the strong and independent association between knowledge and practice. Respondents with satisfactory knowledge had substantially higher odds of demonstrating satisfactory HDC care practices. This supports the central role of knowledge as a determinant of health behavior and aligns with established theoretical models [37,38]. The observed strong association between knowledge and practice should be interpreted with caution. The use of dichotomized composite scores may amplify the effect estimates [39], and residual measurement overlap between constructs may partially explain the magnitude of this association. Nonetheless, the consistency and strength of this relationship emphasize the importance of strengthening patient education as a key intervention target [32].

In the bivariate analysis, employment status and vascular access type were associated with practice, while female sex and shorter duration on hemodialysis showed borderline associations. However, none of these variables remained significant after adjustment, suggesting that their effects may be mediated through knowledge [40]. A borderline association with vascular access type in the adjusted model suggests that patients with permanent catheters may have better practices, possibly due to increased interaction with healthcare providers and repeated counseling [27,31], although this requires further investigation.

Overall, these findings indicate that knowledge was the strongest association factor with HDC care practices in this study population, outweighing the influence of socio-demographic and clinical factors. This has important implications for intervention design, suggesting that efforts to improve patient outcomes should prioritize strengthening knowledge through structured, continuous, and behavior-focused education programs.

This study has several limitations. The cross-sectional nature of the study limits causal interpretation of the relationship between knowledge and practice, as it is not possible to establish directionality between exposure and outcome. The relatively small sample size and single-center setting at a tertiary hospital may limit the external validity of the findings. Therefore, caution is required when generalizing results to other dialysis populations in different settings. Practice was measured using self-reported data, which may be affected by recall and social desirability bias. This may have resulted in overestimation of adherence to recommended catheter care practices. The use of an 80% cutoff to dichotomize knowledge and practice may have reduced variability and potentially inflated observed associations. Additionally, although the questionnaire demonstrated good internal consistency and was reviewed for content validity, formal construct validation (e.g., factor analysis or assessment of construct independence) was not conducted. Given that knowledge and practice were measured using a single self-reported instrument with conceptually related items, some degree of measurement overlap between constructs cannot be excluded, which may have contributed to an overestimation of the observed association between knowledge and practice. Finally, facility-level factors, such as the quality and frequency of patient education, were not assessed and may influence both knowledge and practice.

## Conclusion

In conclusion, this study found that knowledge of HDC care among patients receiving maintenance hemodialysis was moderate, while reported catheter care practices remained suboptimal. Female sex and shorter duration on hemodialysis were associated with better knowledge. Respondents with satisfactory knowledge were more likely to report satisfactory HDC care practices. These findings highlight the potential importance of continuous patient education and reinforcement of practical catheter care skills within routine dialysis services. Further multicenter and longitudinal studies using more robust assessment approaches are recommended to better understand factors influencing HDC care practices and related outcomes.

## Supporting information

S1 File Dataset used in the study. (XLSX)

S2 File Questionnaire. (DOCX)
