# Supplementary material for: Knowledge and practice of hemodialysis catheter care and associated factors among patients on maintenance hemodialysis: An analytical cross-sectional study
Source: PLoS One. 2026 Jul 14;21(7):e0353737. doi: 10.1371/journal.pone.0353737 (PMC13367740; doi:10.1371/journal.pone.0353737)
Supplement: S2 File — (DOCX) [file pone.0353737.s002.docx]

**QUESTIONNAIRE:**

**Factors Associated with Knowledge and Practice Regarding Hemodialysis Catheter Care Among Patients at Bugando Medical Centre in Mwanza, Tanzania**

ID No: _____________________

Date: _____________________

# Questionnaire (English Version)

## Section A: Demographic Characteristics

Instructions: Put a tick (√) in the correct response.

1. What is your age? _____________________
2. Sex

a) Female

b) Male

1. 3. Place of residence

a) Rural

b) Urban

1. 4. What is your marital status?

a) Single

b) Married

c) Divorced

d) Widowed

1. Educational level

a) Illiterate

b) Primary school

c) Secondary/High school

d) University student/graduate

1. Occupational status

a) Unemployed

b) Self-employed

c) Private employee

d) State employee

e) Retired

1. Monthly household income

a) Low (< $500/month)

b) Medium ($500–$1,500/month)

c) High (> $1,500/month)

1. Vascular access (Central venous catheter)

a) Jugular

b) Femoral

c) Permcath

1. Hemodialysis frequency per week

a) 2 times a week

b) 3 times a week

1. Session duration (hours) _____________________
2. Duration on hemodialysis (years)

a) < 1

b) 1–2

c) 2–5

d) > 5

## Section B: Knowledge on Hemodialysis Catheter Care

Instructions: Please read each question carefully and circle the most appropriate answer.

1. What is the primary purpose of a haemodialysis catheter?

a) To give medication

b) To draw blood samples

c) To allow blood flow for dialysis treatment

d) To check blood pressure

1. Where is a haemodialysis catheter commonly inserted?

a) In the arm

b) In the neck, chest, or groin

c) In the leg

d) In the abdomen

1. Which of the following is the correct way to keep your catheter site clean?

a) Wash with soap and water only when it looks dirty

b) Cover the site with a cloth

c) Keep it dry and covered with a sterile dressing at all times

d) Apply lotion around the site

1. Why is it important not to get the catheter site wet?

a) It causes skin itching

b) It may lead to poor blood flow

c) It increases the risk of infection

d) It damages the catheter

1. How often should the catheter dressing be changed?

a) Once a week

b) Only when it falls off

c) After every dialysis session or when wet/dirty

d) Once a month

1. What should you do if you notice redness, swelling, or pus at the catheter site?

a) Wait to see if it goes away

b) Cover it with a bandage yourself

c) Inform the dialysis nurse or doctor immediately

d) Clean it at home with alcohol

1. Can you shower with a haemodialysis catheter?

a) Yes, without covering it

b) No, never

c) Yes, only if it is covered properly and kept dry

d) Yes, if the catheter is inside the arm

1. Why should you avoid using the catheter arm for blood pressure checks or blood draws?

a) It may cause discomfort

b) It may damage the catheter or affect blood flow

c) It makes the catheter fall out

d) There is no need to avoid it

1. What is a sign of catheter malfunction during dialysis?

a) Feeling sleepy

b) Bleeding from the nose

c) Low blood flow or alarms during the session

d) Increase in appetite

1. Who is responsible for caring for your catheter site during dialysis?

a) The patient alone

b) Any family member

c) The dialysis nurse and patient together

d) The doctor only

1. What can happen if the catheter is not cared for properly?

a) Faster dialysis

b) Catheter may work better

c) Increased risk of infection and complications

d) Nothing happens

1. Why should you avoid lifting heavy objects with the catheter arm?

a) It will cause muscle cramps

b) It may lead to catheter dislodgement or poor flow

c) It makes the catheter stronger

d) No reason

## Section C: Practices for Hemodialysis Catheter Care

Instructions: Please indicate how often you perform the following practices related to your haemodialysis catheter care by ticking the most appropriate response.

|  | **Statement** | **1=Never** | **2=Rarely** | **3=Sometimes** | **4=Often** | **5=Always** |
| --- | --- | --- | --- | --- | --- | --- |
|  | Do you wash your hands with soap and water before touching your catheter area? |  |  |  |  |  |
|  | Do you use hand sanitizer when soap and water are not available before touching your catheter? |  |  |  |  |  |
|  | Do you avoid touching your catheter site unless it is necessary? |  |  |  |  |  |
|  | Do you keep your catheter site clean and dry at all times? |  |  |  |  |  |
|  | Do you apply creams or ointments to your catheter site without a doctor’s instruction? |  |  |  |  |  |
|  | Do you change your catheter dressing regularly as advised by healthcare providers? |  |  |  |  |  |
|  | Do you report immediately if your catheter site is red, swollen, or has discharge? |  |  |  |  |  |
|  | Do you cover your catheter site to keep it dry when bathing or showering? |  |  |  |  |  |
|  | Do you avoid swimming or soaking your body in water? |  |  |  |  |  |
|  | Do you avoid wearing tight clothing over the catheter site? |  |  |  |  |  |
|  | Do you come to dialysis sessions with a clean and dry catheter dressing? |  |  |  |  |  |
|  | Do you follow all instructions given by the dialysis staff during your treatment? |  |  |  |  |  |
|  | Do you tell the nurse or doctor if you feel pain or discomfort around your catheter during dialysis? |  |  |  |  |  |
|  | Do you know the signs of infection or problems around your catheter? |  |  |  |  |  |
|  | Do you report fever, chills, or other symptoms that might suggest a catheter infection? |  |  |  |  |  |
