# Supplementary material for: Knowledge and practice of hemodialysis catheter care and associated factors among patients on maintenance hemodialysis: An analytical cross-sectional study
Source: PLoS One. 2026 Jul 14;21(7):e0353737. doi: 10.1371/journal.pone.0353737 (PMC13367740; doi:10.1371/journal.pone.0353737)
Supplement: Attachment — Submitted filename: Response to Reviewers.docx [file pone.0353737.s004.docx]

**Knowledge and Practice of Hemodialysis Catheter Care and Associated Factors Among Patients on Maintenance Hemodialysis: An Analytical Cross-sectional Study**

**Response to reviewer’s comments**

We would like to thank the reviewer for the comments provided to improve this study. We have revised the manuscript as shown in the point-to-point responses to reviewer’s comments provided in the table below. Please let us know if there are any revisions requiring further clarification.

| **Comments** | **Authors Response** |
| --- | --- |
| **Journal requirements:** |  |
| 1. Please ensure that your manuscript meets PLOS ONE's style requirements, including those for file naming. The PLOS ONE style templates can be found at   https://journals.plos.org/plosone/s/file?id=wjVg/PLOSOne_formatting_sample_main_body.pdf and  https://journals.plos.org/plosone/s/file?id=ba62/PLOSOne_formatting_sample_title_authors_affiliations.pdf | Thank you for the comment. Using the provided links, we have revised the manuscript to align with PLOS ONE’s style requirements |
| 2. We note that your Data Availability Statement is currently as follows: All relevant data are within the manuscript and in Supporting Information files.  Please confirm at this time whether or not your submission contains all raw data required to replicate the results of your study. Authors must share the “minimal data set” for their submission. PLOS defines the minimal data set to consist of the data required to replicate all study findings reported in the article, as well as related metadata and methods (https://journals.plos.org/plosone/s/data-availability#loc-minimal-data-set-definition). | We confirm that the submission contains the minimal data set required to replicate all findings reported in the manuscript. All relevant raw data underlying the results, including values used for statistical analyses, tables, and figures, have been provided within the manuscript and Supporting Information files. The submitted data set includes the variables and metadata necessary to reproduce the analyses and study findings in accordance with the PLOS ONE Data Availability policy. |
| 3. We are unable to open your Supporting Information file “S1 Dataset.sav”. Please kindly revise as necessary and re-upload.  If the reviewer comments include a recommendation to cite specific previously published works, please review and evaluate these publications to determine whether they are relevant and should be cited. There is no requirement to cite these works unless the editor has indicated otherwise. | Thank you for bringing this to our attention. We have revised (converted into excel file format) and re-uploaded the Supporting Information file “S1 Dataset.xlsx” to ensure accessibility and compatibility. We confirm that the updated file can now be opened successfully and contains the raw data underlying the study findings. |
| **Additional Editor’s Comments** |  |
| Both reviewers recognized the clinical relevance of the topic and the potential importance of continuous patient education in hemodialysis catheter care. However, important methodological concerns were raised that need to be addressed before the manuscript can be considered further.  In particular, the conceptual overlap between “knowledge” and “practice,” the limitations of the cross-sectional design, insufficient validation details of the questionnaire, and the reliance on self-reported practice measures require more cautious interpretation and clearer methodological justification.  Please revise the manuscript accordingly and provide a detailed point-by-point response to all reviewer comments. | We thank the Editor and Reviewers for their valuable and constructive comments. We appreciate the recognition of the clinical relevance of our study and the importance of continuous patient education in hemodialysis catheter care. We have carefully revised the manuscript to address all concerns raised.  Specifically, we have clarified the conceptual distinction between “knowledge” and “practice” within the study framework and revised the discussion to ensure a more cautious interpretation of their relationship. We have also acknowledged the inherent limitations of the cross-sectional study design, particularly regarding causal inference, within the limitations section.  In addition, further details regarding the development, validation, and reliability assessment of the questionnaire have been incorporated into the Methods section to strengthen methodological transparency. We have also clarified the use of self-reported practice measures and discussed the potential for reporting bias as a study limitation.  A detailed point-by-point response to all reviewer comments has been prepared and submitted alongside the revised manuscript. We believe these revisions have substantially improved the clarity, rigor, and overall quality of the manuscript. |
|  |  |
| **Reviewer #1** |  |
| For effective care of hemodialysis catheters, the authors investigated the gap between  patient knowledge and practice, using a structured questionnaire. Regarding practice, knowledge was the only independent predictor. The strengths of this paper are twofold. First, it evaluated the importance of continuous education based on the facts that shorter duration on hemodialysis was independently associated with better knowledge. Second, it clearly identified areas of weakness in both knowledge and practice. There are no problems with the methodology or discussion. This paper deserves to be accepted. | We thank the reviewer for the positive and encouraging comments. We appreciate the recognition of the clinical relevance of our study, the importance of continuous patient education, and the value of identifying gaps in patients’ knowledge and practice regarding hemodialysis catheter care. |
| **Reviewer #2** |  |
| This manuscript addresses an important clinical issue regarding knowledge and practice of hemodialysis catheter care among patients receiving maintenance hemodialysis. Catheter-related complications remain a major challenge, particularly in resource-limited settings, and patient education is undoubtedly an important component of prevention strategies. | We thank the reviewer for recognizing the clinical importance of our study and the relevance of patient education in preventing hemodialysis catheter-related complications, particularly in resource-limited settings. |
| However, the study has several important methodological and conceptual limitations that substantially weaken the validity and interpretability of the findings. In particular, the insufficient distinction between “knowledge” and “practice,” the limitations inherent to the cross-sectional design, concerns regarding questionnaire validity, and the reliance on self-reported practice measures significantly limit confidence in the central conclusions. In addition, the relatively small single-center sample further restricts the robustness and generalizability of the results. | We acknowledge the reviewer’s concerns regarding the methodological and conceptual limitations of the study. The distinction between knowledge and practice has been clarified in the revised manuscript, and the limitations of the cross-sectional design, use of self-reported measures, and questionnaire validity have been explicitly addressed in the Methods and Discussion sections. We have also highlighted the constraints related to the single-center and relatively small sample size, and their implications for the generalizability of the findings. |
| While the topic is clinically relevant, the current manuscript does not provide sufficiently rigorous evidence to support its main conclusions in its present form. | We thank the reviewer for this important comment. In response, we revised the manuscript to strengthen the methodological clarity and interpretation of findings by clarifying the distinction between knowledge and practice, expanding the description of questionnaire validation, and acknowledging the limitations related to the cross-sectional design, self-reported measures, and single-center sample. We also revised the Discussion and Conclusion sections to provide a more cautious interpretation of the findings. |
| 1. Conceptual overlap between “knowledge” and “practice”  The most important limitation of this study is the insufficient separation between the constructs of “knowledge” and “practice.” Several practice items appear to directly reflect knowledge-based behaviors, such as recognizing infection signs, reporting symptoms, and following healthcare instructions. This conceptual overlap likely inflated the observed association between knowledge and practice (AOR 47.39). Without formal assessment of construct independence (e.g., factor analysis or validation analysis), the conclusion that knowledge independently predicts practice is not adequately supported. | We thank the reviewer for this important comment. We acknowledge the concern regarding conceptual overlap between knowledge and practice. The manuscript has been revised to clarify the distinction between these constructs and to provide a more cautious interpretation of their relationship. We also acknowledge that formal assessment of construct independence was not performed, and this has been stated as a limitation. The conclusion regarding knowledge as a predictor of practice has been tempered accordingly. |
| 2. Cross-sectional design cannot support directional interpretation  The manuscript repeatedly implies that improving knowledge would improve catheter care practice. However, because this is a cross-sectional study, causal or directional relationships cannot be inferred. Reverse causation is also plausible, whereby patients with better practices may acquire greater knowledge through increased engagement with healthcare providers. The interpretation throughout the Discussion and Conclusion should therefore be substantially tempered. | We thank the reviewer for this important methodological clarification. We acknowledge that the cross-sectional design does not allow inference of causal or directional relationships between knowledge and practice. The manuscript has been revised to remove causal wording and to temper interpretations throughout the Discussion and Conclusion. We also recognize the possibility of reverse causation, which has been reflected in the revised limitations and interpretation of findings. |
| 3. Questionnaire validity is insufficiently demonstrated  Although Cronbach’s alpha is reported, internal consistency alone is insufficient to establish questionnaire validity. The manuscript lacks adequate information regarding construct validity, item development, translation validation, and potential redundancy between items. Furthermore, the questionnaire itself is not provided as supplementary material, limiting reproducibility and external evaluation of the measurement tool. | We thank the reviewer for this important methodological observation. We acknowledge that Cronbach’s alpha alone does not fully establish questionnaire validity. The manuscript has been revised to clarify the tool development process, including expert review and pretesting, and to acknowledge that formal construct validation and translation validation procedures were not conducted. We also note that the questionnaire has now been included as supplementary material to enhance transparency and reproducibility. These revisions strengthen the reporting of the measurement tool and its limitations. |
| 4. Self-reported practice may substantially overestimate adherence  Practice was assessed entirely through self-reported responses collected during dialysis sessions in the presence of healthcare personnel. This creates a substantial risk of social desirability bias and may lead to overestimation of appropriate catheter care behaviors. The absence of objective observational measures further limits confidence in the reported practice outcomes. | We thank the reviewer for this important methodological concern. We acknowledge that self-reported practice measures are subject to social desirability and recall bias, particularly given the clinical setting in which data were collected. The manuscript has been revised to explicitly recognize this limitation and to temper the interpretation of practice-related findings. We also note the absence of objective observational assessment as a limitation affecting the robustness of practice measurement. |
| 5. Small single-center sample limits reliability and generalizability  This study included only 97 participants from a single center. Such a sample size may be insufficient for stable multivariable logistic regression analysis with multiple predictors. The limited sample and single-center design also substantially reduce the external validity and generalizability of the findings to broader hemodialysis populations. | We thank the reviewer for this important methodological point. We acknowledge that the relatively small sample size and single-center design may limit the stability of multivariable estimates and reduce the generalizability of the findings. This limitation has been explicitly stated in the revised manuscript, and the interpretation of results has been appropriately tempered to reflect these constraints. |
| Although the study addresses a clinically meaningful topic, the current manuscript has substantial methodological and conceptual weaknesses that limit confidence in the findings and their interpretation. In particular, the overlap between the “knowledge” and “practice” constructs and the limitations of the measurement methodology require major reconsideration.  A substantially revised manuscript with clearer construct validation, more cautious interpretation, and stronger methodological justification would be necessary before the study could be considered for publication. | We thank the reviewer for this comprehensive assessment and for acknowledging the clinical importance of the study topic. We have substantially revised the manuscript to address the methodological and conceptual concerns raised, including clarifying the distinction between knowledge and practice, strengthening the description and justification of the measurement approach, and explicitly acknowledging limitations related to construct validation, self-reported data, and study design. We have also revised the Discussion and Conclusion to ensure a more cautious interpretation of the findings. We appreciate the guidance provided, which has helped improve the rigor and clarity of the manuscript. |
