# Supplementary material for: Knowledge and practice of hemodialysis catheter care and associated factors among patients on maintenance hemodialysis: An analytical cross-sectional study
Source: PLoS One. 2026 Jul 14;21(7):e0353737. doi: 10.1371/journal.pone.0353737 (PMC13367740; doi:10.1371/journal.pone.0353737)
Supplement: Peer review history

# Peer review: Decision Letter 0

**DOI:** 10.1371/journal.pone.0353737.r001

**Authors:** Yoshitaka Ishibashi

22 May 2026

PONE-D-26-19552

Knowledge and Practice of Hemodialysis Catheter Care and Associated Factors Among Patients on Maintenance Hemodialysis: An Analytical Cross-sectional Study

PLOS One

Dear Dr. Ambikile,

Thank you for submitting your manuscript to PLOS ONE. After careful consideration, we feel that it has merit but does not fully meet PLOS ONE’s publication criteria as it currently stands. Therefore, we invite you to submit a revised version of the manuscript that addresses the points raised during the review process.

Please submit your revised manuscript by Jul 06 2026 11:59PM. If you will need more time than this to complete your revisions, please reply to this message or contact the journal office at plosone@plos.org. When you're ready to submit your revision, log on to https://www.editorialmanager.com/pone/ and select the 'Submissions Needing Revision' folder to locate your manuscript file.

Please include the following items when submitting your revised manuscript:

- A letter that responds to each point raised by the academic editor and reviewer(s). You should upload this letter as a separate file labeled 'Response to Reviewers'.
- A marked-up copy of your manuscript that highlights changes made to the original version. You should upload this as a separate file labeled 'Revised Manuscript with Track Changes'.
- An unmarked version of your revised paper without tracked changes. You should upload this as a separate file labeled 'Manuscript'.

If you would like to make changes to your financial disclosure, please include your updated statement in your cover letter. Guidelines for resubmitting your figure files are available below the reviewer comments at the end of this letter.

If applicable, we recommend that you deposit your laboratory protocols in protocols.io to enhance the reproducibility of your results. Protocols.io assigns your protocol its own identifier (DOI) so that it can be cited independently in the future. For instructions see: https://journals.plos.org/plosone/s/submission-guidelines#loc-laboratory-protocols. Additionally, PLOS ONE offers an option for publishing peer-reviewed Lab Protocol articles, which describe protocols hosted on protocols.io. Read more information on sharing protocols at https://plos.org/protocols?utm_medium=editorial-email&utm_source=authorletters&utm_campaign=protocols.

As the corresponding author, your ORCID iD is verified in the submission system and will appear in the published article. PLOS supports the use of ORCID, and we encourage all coauthors to register for an ORCID iD and use it as well. Please encourage your coauthors to verify their ORCID iD within the submission system before final acceptance, as unverified ORCID iDs will not appear in the published article. *Only*  the individual author can complete the verification step; PLOS staff *cannot*  verify ORCID iDs on behalf of authors.

We look forward to receiving your revised manuscript.

Kind regards,

Yoshtiaka Ishibashi

Academic Editor

PLOS ONE

Journal Requirements:

When submitting your revision, we need you to address these additional requirements.

1.Please ensure that your manuscript meets PLOS ONE's style requirements, including those for file naming. The PLOS ONE style templates can be found at

https://journals.plos.org/plosone/s/file?id=wjVg/PLOSOne_formatting_sample_main_body.pdf and

https://journals.plos.org/plosone/s/file?id=ba62/PLOSOne_formatting_sample_title_authors_affiliations.pdf

2. We note that your Data Availability Statement is currently as follows: All relevant data are within the manuscript and in Supporting Information files.

Please confirm at this time whether or not your submission contains all raw data required to replicate the results of your study. Authors must share the “minimal data set” for their submission. PLOS defines the minimal data set to consist of the data required to replicate all study findings reported in the article, as well as related metadata and methods (https://journals.plos.org/plosone/s/data-availability#loc-minimal-data-set-definition).

For example, authors should submit the following data:

- The values behind the means, standard deviations and other measures reported;

- The values used to build graphs;

- The points extracted from images for analysis.

Authors do not need to submit their entire data set if only a portion of the data was used in the reported study.

If your submission does not contain these data, please either upload them as Supporting Information files or deposit them to a stable, public repository and provide us with the relevant URLs, DOIs, or accession numbers. For a list of recommended repositories, please see https://journals.plos.org/plosone/s/recommended-repositories.

If there are ethical or legal restrictions on sharing a de-identified data set, please explain them in detail (e.g., data contain potentially sensitive information, data are owned by a third-party organization, etc.) and who has imposed them (e.g., an ethics committee). Please also provide contact information for a data access committee, ethics committee, or other institutional body to which data requests may be sent. If data are owned by a third party, please indicate how others may request data access.

3. We are unable to open your Supporting Information file “S1 Dataset.sav”. Please kindly revise as necessary and re-upload.

If the reviewer comments include a recommendation to cite specific previously published works, please review and evaluate these publications to determine whether they are relevant and should be cited. There is no requirement to cite these works unless the editor has indicated otherwise.

Additional Editor Comments:

Dear Authors,

Thank you for submitting your manuscript to PLOS ONE.

Both reviewers recognized the clinical relevance of the topic and the potential importance of continuous patient education in hemodialysis catheter care. However, important methodological concerns were raised that need to be addressed before the manuscript can be considered further.

In particular, the conceptual overlap between “knowledge” and “practice,” the limitations of the cross-sectional design, insufficient validation details of the questionnaire, and the reliance on self-reported practice measures require more cautious interpretation and clearer methodological justification.

Please revise the manuscript accordingly and provide a detailed point-by-point response to all reviewer comments.

I look forward to receiving your revised manuscript.

Sincerely,

Yoshtiaka Ishibashi

Academic Editor

PLOS ONE

[Note: HTML markup is below. Please do not edit.]

Reviewers' comments:

Reviewer's Responses to Questions

**Comments to the Author**

1. Is the manuscript technically sound, and do the data support the conclusions?

The manuscript must describe a technically sound piece of scientific research with data that supports the conclusions. Experiments must have been conducted rigorously, with appropriate controls, replication, and sample sizes. The conclusions must be drawn appropriately based on the data presented.

Reviewer #1: Yes

Reviewer #2: Partly

**********

2. Has the statistical analysis been performed appropriately and rigorously?

Reviewer #1: Yes

Reviewer #2: No

**********

3. Have the authors made all data underlying the findings in their manuscript fully available?

The PLOS Data policy requires authors to make all data underlying the findings described in their manuscript fully available without restriction, with rare exception (please refer to the Data Availability Statement in the manuscript PDF file). The data should be provided as part of the manuscript or its supporting information, or deposited to a public repository. For example, in addition to summary statistics, the data points behind means, medians and variance measures should be available. If there are restrictions on publicly sharing data—e.g. participant privacy or use of data from a third party—those must be specified.

Reviewer #1: Yes

Reviewer #2: Yes

**********

4. Is the manuscript presented in an intelligible fashion and written in standard English?

PLOS ONE does not copyedit accepted manuscripts, so the language in submitted articles must be clear, correct, and unambiguous. Any typographical or grammatical errors should be corrected at revision, so please note any specific errors here.

Reviewer #1: Yes

Reviewer #2: Yes

**********

5. Review Comments to the Author

Please use the space provided to explain your answers to the questions above. You may also include additional comments for the author, including concerns about dual publication, research ethics, or publication ethics. (Please upload your review as an attachment if it exceeds 20,000 characters)

Reviewer #1: For effective care of hemodialysis catheters, the authors investigated the gap between

patient knowledge and practice, using a structured questionnaire. Regarding practice, knowledge was the only independent predictor. The strengths of this paper are twofold. First, it evaluated the importance of continuous education based on the facts that shorter duration on hemodialysis was independently associated with better knowledge. Second, it clearly identified areas of weakness in both knowledge and practice. There are no problems with the methodology or discussion. This paper deserves to be accepted.

Reviewer #2: This manuscript addresses an important clinical issue regarding knowledge and practice of hemodialysis catheter care among patients receiving maintenance hemodialysis. Catheter-related complications remain a major challenge, particularly in resource-limited settings, and patient education is undoubtedly an important component of prevention strategies.

However, the study has several important methodological and conceptual limitations that substantially weaken the validity and interpretability of the findings. In particular, the insufficient distinction between “knowledge” and “practice,” the limitations inherent to the cross-sectional design, concerns regarding questionnaire validity, and the reliance on self-reported practice measures significantly limit confidence in the central conclusions. In addition, the relatively small single-center sample further restricts the robustness and generalizability of the results.

While the topic is clinically relevant, the current manuscript does not provide sufficiently rigorous evidence to support its main conclusions in its present form.

1. Conceptual overlap between “knowledge” and “practice”

The most important limitation of this study is the insufficient separation between the constructs of “knowledge” and “practice.” Several practice items appear to directly reflect knowledge-based behaviors, such as recognizing infection signs, reporting symptoms, and following healthcare instructions. This conceptual overlap likely inflated the observed association between knowledge and practice (AOR 47.39). Without formal assessment of construct independence (e.g., factor analysis or validation analysis), the conclusion that knowledge independently predicts practice is not adequately supported.

2. Cross-sectional design cannot support directional interpretation

The manuscript repeatedly implies that improving knowledge would improve catheter care practice. However, because this is a cross-sectional study, causal or directional relationships cannot be inferred. Reverse causation is also plausible, whereby patients with better practices may acquire greater knowledge through increased engagement with healthcare providers. The interpretation throughout the Discussion and Conclusion should therefore be substantially tempered.

3. Questionnaire validity is insufficiently demonstrated

Although Cronbach’s alpha is reported, internal consistency alone is insufficient to establish questionnaire validity. The manuscript lacks adequate information regarding construct validity, item development, translation validation, and potential redundancy between items. Furthermore, the questionnaire itself is not provided as supplementary material, limiting reproducibility and external evaluation of the measurement tool.

4. Self-reported practice may substantially overestimate adherence

Practice was assessed entirely through self-reported responses collected during dialysis sessions in the presence of healthcare personnel. This creates a substantial risk of social desirability bias and may lead to overestimation of appropriate catheter care behaviors. The absence of objective observational measures further limits confidence in the reported practice outcomes.

5. Small single-center sample limits reliability and generalizability

This study included only 97 participants from a single center. Such a sample size may be insufficient for stable multivariable logistic regression analysis with multiple predictors. The limited sample and single-center design also substantially reduce the external validity and generalizability of the findings to broader hemodialysis populations.

Although the study addresses a clinically meaningful topic, the current manuscript has substantial methodological and conceptual weaknesses that limit confidence in the findings and their interpretation. In particular, the overlap between the “knowledge” and “practice” constructs and the limitations of the measurement methodology require major reconsideration.

A substantially revised manuscript with clearer construct validation, more cautious interpretation, and stronger methodological justification would be necessary before the study could be considered for publication.

**********

6. PLOS authors have the option to publish the peer review history of their article (what does this mean?). If published, this will include your full peer review and any attached files.

If you choose “no”, your identity will remain anonymous but your review may still be made public.

**Do you want your identity to be public for this peer review?**  For information about this choice, including consent withdrawal, please see our Privacy Policy.

Reviewer #1: No

Reviewer #2: No

**********

[NOTE: If reviewer comments were submitted as an attachment file, they will be attached to this email and accessible via the submission site. Please log into your account, locate the manuscript record, and check for the action link "View Attachments". If this link does not appear, there are no attachment files.]

To ensure your figures meet our technical requirements, please review our figure guidelines: https://journals.plos.org/plosone/s/figures

You may also use PLOS’s free figure tool, NAAS, to help you prepare publication quality figures: https://journals.plos.org/plosone/s/figures#loc-tools-for-figure-preparation.

NAAS will assess whether your figures meet our technical requirements by comparing each figure against our figure specifications.

# Author comment: Author response to Decision Letter 1

**DOI:** 10.1371/journal.pone.0353737.r002

26 May 2026

We have revised the manuscript based on the reviewers and editor's comments

Attachment Submitted filename: Response to Reviewers.docx

# Peer review: Decision Letter 1

**DOI:** 10.1371/journal.pone.0353737.r003

**Authors:** Yoshitaka Ishibashi, Yoshitaka Ishibashi

29 Jun 2026

Knowledge and Practice of Hemodialysis Catheter Care and Associated Factors Among Patients on Maintenance Hemodialysis: An Analytical Cross-sectional Study

PONE-D-26-19552R1

Dear Dr. Ambikile,

Both reviewers now find the manuscript substantially improved, and the major methodological concerns raised during the previous review round have been adequately addressed. Reviewer 2 raises only a minor remaining concern regarding the use of causal language when describing the relationship between knowledge and practice. I agree with this comment. Given the cross-sectional design of the study, the conclusions should consistently describe this relationship as an association rather than implying causality. This is a minor wording issue that can be readily addressed without further external review. Subject to these minor textual revisions, I consider the manuscript suitable for publication.

Within one week, you’ll receive an e-mail detailing the required amendments. When these have been addressed, you’ll receive a formal acceptance letter and your manuscript will be scheduled for publication.

An invoice will be generated when your article is formally accepted. Please note, if your institution has a publishing partnership with PLOS and your article meets the relevant criteria, all or part of your publication costs will be covered. Please make sure your user information is up-to-date by logging into Editorial Manager at Editorial Manager® and clicking the ‘Update My Information' link at the top of the page. For questions related to billing, please contact billing support.

If your institution or institutions have a press office, please notify them about your upcoming paper to help maximize its impact. If they’ll be preparing press materials, please inform our press team as soon as possible -- no later than 48 hours after receiving the formal acceptance. Your manuscript will remain under strict press embargo until 2 pm Eastern Time on the date of publication. For more information, please contact onepress@plos.org.

Kind regards,

Yoshitaka Ishibashi

Academic Editor

PLOS One

# Editor report: Acceptance letter

**DOI:** 10.1371/journal.pone.0353737.r004

**Authors:** Yoshitaka Ishibashi, Yoshitaka Ishibashi

PONE-D-26-19552R1

PLOS One

Dear Dr. Ambikile,

I'm pleased to inform you that your manuscript has been deemed suitable for publication in PLOS One. Congratulations! Your manuscript is now being handed over to our production team.

At this stage, our production department will prepare your paper for publication. This includes ensuring the following:

* All references, tables, and figures are properly cited

* All relevant supporting information is included in the manuscript submission,

* There are no issues that prevent the paper from being properly typeset

You will receive further instructions from the production team, including instructions on how to review your proof when it is ready. Please keep in mind that we are working through a large volume of accepted articles, so please give us a few days to review your paper and let you know the next and final steps.

Lastly, if your institution or institutions have a press office, please let them know about your upcoming paper now to help maximize its impact. If they'll be preparing press materials, please inform our press team within the next 48 hours. Your manuscript will remain under strict press embargo until 2 pm Eastern Time on the date of publication. For more information, please contact onepress@plos.org.

You will receive an invoice from PLOS for your publication fee after your manuscript has reached the completed accept phase. If you receive an email requesting payment before acceptance or for any other service, this may be a phishing scheme. Learn how to identify phishing emails and protect your accounts at https://explore.plos.org/phishing.

If we can help with anything else, please email us at customercare@plos.org.

Thank you for submitting your work to PLOS ONE and supporting open access.

Kind regards,

PLOS ONE Editorial Office Staff

on behalf of

Dr. Yoshitaka Ishibashi

Academic Editor

PLOS One
